# Unboxing the network among long non-coding RNAs and TGF-β signaling in cancer

**DOI:** 10.48101/ujms.v129.10614

**Published:** 2024-03-26

**Authors:** Dorival Mendes Rodrigues-Junior, Aristidis Moustakas

**Affiliations:** Department of Medical Biochemistry and Microbiology, Science for Life Laboratory, Uppsala University, Uppsala, Sweden

**Keywords:** Cancer, epigenetics, non-coding RNA, signal transduction, transforming growth factor-β

## Abstract

Deeper analysis of molecular mechanisms arising in tumor cells is an unmet need to provide new diagnostic and therapeutic strategies to prevent and treat tumors. The transforming growth factor β (TGF-β) signaling has been steadily featured in tumor biology and linked to poor prognosis of cancer patients. One pro-tumorigenic mechanism induced by TGF-β is the epithelial-to-mesenchymal transition (EMT), which can initiate cancer dissemination, enrich the tumor stem cell population, and increase chemoresistance. TGF-β signals via SMAD proteins, ubiquitin ligases, and protein kinases and modulates the expression of protein-coding and non-coding RNA genes, including those encoding larger than 500 nt transcripts, defined as long non-coding RNAs (lncRNAs). Several reports have shown lncRNAs regulating malignant phenotypes by directly affecting epigenetic processes, transcription, and post-transcriptional regulation. Thus, this review aims to update and summarize the impact of TGF-β signaling on the expression of lncRNAs and the function of such lncRNAs as regulators of TGF-β signaling, and how these networks might impact specific hallmarks of cancer.

## Introduction

Signal transduction pathways serve as modulators of cellular physiology by acting on diverse systems that maintain cellular function, including the genome, whose expression is modulated, thus generating specific biological adaptations and responses that mediate new cellular behavior (1). For this reason, most signaling pathways become implicated in diseases, including cancer. The mammalian genomes contain a large number of genes, including genes that encode mRNAs and non-coding RNAs, such as, rRNA, tRNA, micro-RNA (miRNA), piwi RNA (piRNA), small nucleolar RNA (snRNA), long non-coding RNA (lncRNA), and more. We here aim at summarizing and critically evaluating evidence on the regulation of expression and function of lncRNAs by the transforming growth factor β (TGF-β) family of signaling pathways.

## TGF-β signaling

**Figure 1:**
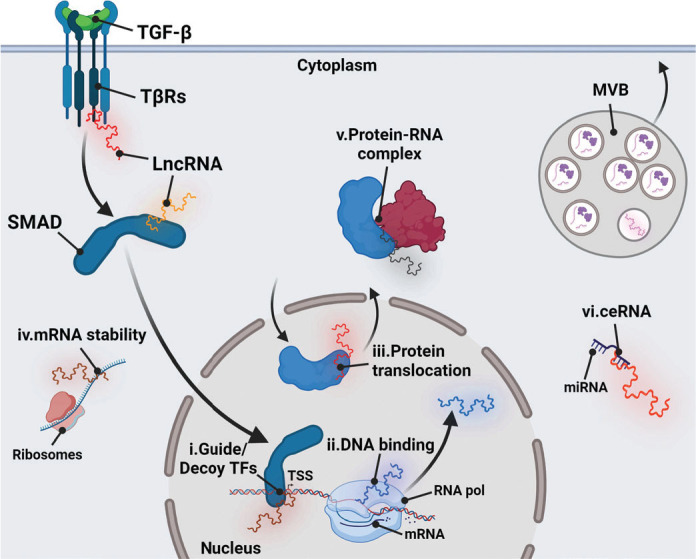
Network of lncRNAs and TGF-β signaling in cancer. TGF-β binds to the type II and type I receptors (TβRs) on the cell surface, signaling via inter-receptor trans-phosphorylation. The type I receptor phosphorylates SMAD2/3, which promotes their oligomerization with SMAD4 (presented for simplicity as SMAD), and in parallel, TGF-β signaling induces RAS, MEK, ERK, and other protein kinase pathways (not shown), that, together with transcription factors (TFs), regulate the expression of lncRNAs (shown as multicolor single-stranded molecules in order to emphasize the diversity of the implicated lncRNA species). According to the respective cellular compartment, lncRNAs modulate TGF-β signaling at different steps. Nuclear lncRNAs act as (i) guide or decoy molecules for TF complexes to promote gene transcription (TSS, transcription start site) (ii) as DNA binding molecules inducing triple helix formations or (iii) facilitate protein translocation from one compartment to another. Cytoplasmic lncRNAs can regulate (iv) mRNA stability, (v) can act as scaffolds, stabilizing protein-RNA complexes, or (vi) can regulate mRNA translation and stability through competitive endogenous RNA (ceRNA) function against microRNAs (miRNAs). LncRNAs are also sorted as cargo in intraluminal vesicles of multivesicular bodies (MVBs), which release their lncRNA content into the extracellular milieu. *Created with BioRender.com*. TGF-β, transforming growth factor β; IncRNA, long non-coding RNAs.

A prominent developmental signaling pathway with strong implications in cancer is that of TGF-β (2), whose molecular constituents attract attention for the development of improved treatment of diverse tumor types (2). The dimeric ligand forms complexes with two similar plasma membrane receptors (type I, TGFβRI, and type II, TGFβRII), whose ligand-mediated protein complex results in the activation of the cytoplasmic protein kinase domain of TGFβRI (2). TGF-β binding to the two signaling receptors is facilitated by association with the heparan-sulfate proteoglycan co-receptor TGFβRIII or betaglycan or other co-receptors, which oligomerize with TGFβRII and TGFβRI (2). The TGFβRI protein kinase phosphorylates the C-terminal di-serine residues in SMAD family proteins, specifically SMAD2 and SMAD3, which then form complexes with SMAD4, thus stabilizing a transcriptional SMAD complex that associates with chromatin and modulates the expression of many different genes (3). The TGF-β receptors associate with other signaling proteins that lead to activation of protein and lipid kinases and of small GTPases (2). Such complementary signaling inputs can post-translationally modify the SMADs, their interacting transcription, or chromatin modulating co-factors, or alternatively, these inputs control the assembly and activity of cytoplasmic or membrane-associated factors that elicit the physiological response of cells to TGF-β (2). TGF-β signaling can also regulate splicing and maturation of specific mRNAs, processing of certain miRNAs, whereas miRNAs and other non-protein coding RNAs, such as lncRNAs, regulate different aspects of the signaling pathway (2, 4), as discussed in detail in this article (see Figure 1).

The initial implication of a role of TGF-β1 in cancer biology was followed by the understanding that most other members of the TGF-β family, for example, the bone morphogenetic proteins (BMPs) and the activins, are also involved in cancer development, and each of these factors exhibits unique and tumor type-specific functions (5). Cancer cell biological research and *in vivo* mouse models of cancer coupled to a large number of studies in cancer patients have suggested with a high degree of confidence that TGF-β is a suppressor of pre-malignant hyperplasia in many solid and hematopoietic tumors, but is also involved in the progression of tumors, especially during the stages of dissemination of tumor cells via local or distant invasion and metastasis (2). The ability of TGF-β to arrest the cell cycle of epithelial, endothelial, hematopoietic, and other cell types contributes to the suppression of premalignant oncogenic tissue growth (2). TGF-β can also induce apoptosis in specific tissue types (e.g. liver and prostate) and thus antagonize tumorigenic development based on TGF-β’s homeostatic functions (2). In a different biological context of tumor development, TGF-β is a potent inducer of epithelial-mesenchymal transition (EMT), a cell differentiation change that promotes the dissemination of tumor cells away from the primary tumor, and a process that is not strictly associated with epithelial tumor cells (in carcinomas), but is also relevant for a variety of other tumors, including brain and hematopoietic tumors (6). TGF-β also acts as pro-stemness factor that helps the survival and maintenance of cancer stem cells (7). Possibly, the most powerful action of TGF-β in cancer is the suppression of immune cell proliferation and differentiation, making TGF-β a very potent agent that helps the long-term survival of tumors and the inactivation of the immune response against the tumor (2).

## Long non-coding RNAs

All genomes contain lncRNA genes, including those whose function was elucidated since the early days of molecular biology (rRNAs and tRNAs) (8). Until the year of 2021, about 20,000 different lncRNAs were described (9). Their majority map at distinct chromosomal regions away from protein-coding genes, yet some lncRNAs are transcribed from the antisense strand of a protein-coding cistron, which may or may not overlap with the mRNA, and other are completely embedded into an intron or alternatively share a common transcriptional promoter with a protein-coding gene (8).

LncRNA genes generate mature RNAs of more than 500 nt length and are normally produced by the same transcriptional, maturation, and splicing mechanisms catalyzed by RNA polymerase II and capping and polyadenylating enzymes as those of mRNAs (8). A major difference and aspect of debate that has been difficult to experimentally resolve is the extent to which open reading frames recognized in the sequence of mature lncRNAs are functionally translated into polypeptides or not. In general, regulatory features of the lncRNA secondary structure are frequently considered as determinants of translatability of lncRNAs (8), whereas studies of direct ribosomal association with lncRNAs and their translation into polypeptides are most frequently bypassed. Accordingly, some previously defined lncRNAs were shown to generate short polypeptides with physiological function, such as the *homeobox B cluster antisense RNA 3* (*HOXB-AS3*), whose 53 amino acid-long polypeptide regulates alternative splicing of the pyruvate kinase M in colorectal cancer (CRC) cells (10). Growing evidence though suggests a more universal function of lncRNAs as components of large ribonucleoprotein assemblies that regulate cell biological processes in the nucleus, on chromatin or in the cytoplasm and even near the plasma membrane (8).

In the context of cancer, lncRNA expression can be misregulated, thus facilitating specific steps in cancer development, partially due to the demonstrated function of lncRNAs in normal stem cell viability and the control of cell differentiation (8). LncRNAs not only function by interacting with proteins but also assemble triple-helices with DNA strands on chromatin and base-pair with other RNAs, including miRNAs, acting as competing endogenous RNAs (ceRNAs) that sequester miRNAs into specific cellular subcompartments (8). All of these mechanisms have been demonstrated to be functionally important in cancer cell biology (see Figure 1). It is therefore interesting and important to research and uncover unknown mechanisms and functional links between lncRNAs and signaling pathways such as TGF-β, which is implicated in all aspects of cancer development. Since the majority of lncRNA genes remain functionally unexplored, this field of research promises exciting future discoveries.

## Breast cancer

Breast cancer (BRCA) was estimated by the World Health Organization (WHO) as one of the most frequent tumors worldwide, reporting 2.3 million new cases in 2020 and 685,000 BRCA-related deaths (11). lncRNAs contribute to our understanding of BRCA biology, and as potential new biomarkers and therapeutic targets to defeat this disease (12). In line with this, the expression of *lncRNA-activated by transforming growth factor beta* (*lncRNA-ATB*), one of the first lncRNAs described to be induced upon TGF-β signaling activation (13), has been correlated with the worst prognosis of BRCA patients and trastuzumab resistance by competing with miRNAs (14). Additionally, circulating *lncRNA-ATB* can be used as a non-invasive diagnostic marker in serum for early stages of BRCA (15). Nevertheless, how TGF-β signaling induces *lncRNA-ATB* expression has been poorly explored. A distinct lncRNA with prognostic potential in several tumors, including BRCA, is the *Smad/Myc coactivator or LOC284454* (*Smyca*) (16). *Smyca* expression can be induced by TGF-β signaling via SMAD, and in triple negative BRCA (TNBC) cells, *Smyca* promoted TGF-β/SMAD signaling by binding to the MH1 domains of SMAD3 and SMAD4, but not SMAD2, thus increasing the stability of the SMAD3/4 complex, leading to the expression of TGF-β responsive genes, responsible for EMT, cell motility, and drug resistance. Moreover, *Smyca* bound to c-Myc independently of SMAD3/4. Interestingly, the balance promoted by c-Myc and SMAD3/4 in competing for this lncRNA was shown to modulate glycolysis (16). Of note, after activation of TGF-β signaling, the SMAD complex can be formed either in the cytosol or in the nucleus (2), and *Smyca* was characterized as a nuclear lncRNA. Since binding of *Smyca* with SMAD3 or SMAD4 was described without activating TGF-β signaling, probably *Smyca* can bind to non-active SMADs shuttling in the nucleus. Notwithstanding, this study described novel mechanisms about a lncRNA stabilizing the TGF-β signaling protein complex in BRCA.

Additional lncRNAs reported as potentially poor prognostic markers for TNBC patients were the *tumor suppressor candidate 7* (*TUSC7*) and *ADAMTS9 antisense RNA 2* (*ADAMTS9-AS2*) (17, 18). *TUSC7* can bind to *miR-1224-3p*, and its overexpression decreased cell motility and promoted the sensitivity of MDA-MB-468 cells to cytotoxic drugs, while *TUSC7* knockdown in MDA-MB-231 cells enhanced *TGFBR2* levels, suggesting a negative role of *TUSC7* on TGF-β signaling (18). Furthermore, *ADAMTS9-AS2* can interact with and modulate the expression of the ribosomal protein L22 (RPL22) (17). These data were further correlated with the translational downregulation of *TGFBRI* and *SMAD2*, thereby modulating TGF-β signaling negatively. RPL22 can bind to intronic sequences in the *SMAD2* pre-mRNA, inducing exon 9 skipping, which reduced SMAD2 protein levels (19). Additionally, *ADAMTS9-AS2* overexpression suppressed proliferation and invasion of TNBC cells in culture and *in vivo*, which is consistent with TGF-β signaling controlling TNBC progression (17). Nevertheless, mechanistically, how *ADAMTS9-AS2* interacting with RPL22 could negatively modulate *TGFBR1* expression, and how *TUSC7* and *ADAMTS9-AS2* expressions could be regulated were not explored. Conversely to *TUSC7* and *ADAMTS9-AS2*, the loss of function assays of *thymopoietin antisense transcript 1* (*TMPO-AS1*) in MDA-MB-231 cells revealed a reduction of gene signatures related to TGF-β signaling, as shown by *TGFBR1* and *TGFBR2* expression, in comparison to the cells expressing *TMPO-AS1* (20). SiRNA-based treatment against *TMPO-AS1* impaired proliferation and migration of TNBC using in culture and *in vivo* models (20). Furthermore, the activation of TGF-β signaling in BRCA cells induced the expression of the lncRNA *urothelial cancer associated 1* (*UCA1*) (21), and its expression was further associated with doxorubicin resistance. However, mechanistically, how can *TMPO-AS1* modulate *TGFBR1* and *TGFBR2* expressions, and how can *UCA1* promote doxorubicin resistance apart from inducing TGF-β signaling was not elucidated.

A novel lncRNA positively regulating TGF-β signaling is the *lncRNA enforcing TGF-β signaling 1* (*LETS1*) (22). *LETS1* is a nuclear lncRNA induced by TGF-β–SMAD signaling that induced EMT and migration in breast and lung cancer cells. RNA immunoprecipitation (RIP) and RNA pull-down assays revealed a protein-RNA complex among *LETS1* and the nuclear factor of activated T cells (NFAT5), thus *LETS1* enhancing NFAT5 binding to the orphan *nuclear receptor 4A1* (*NR4A1*) promoter. It is important to mention that NR4A1 formed protein complexes with AXIN2 and RNF12 or ARKADIA to facilitate SMAD7 polyubiquitination and its subsequent degradation (23). Consequently, this process initiated by *LETS1* could inhibit TGFβRI polyubiquitination via SMAD7, thus establishing a positive feedback loop for TGF-β–SMAD signaling.

Of importance, since lncRNAs also regulate biological responses through encoding micropeptides, the *long intergenic non-protein coding RNA 665* (*LINC00665*) could be translated to the CIP2A binding peptide (CIP2A-BP, a 52-amino acid peptide), whose translation was downregulated after TGF-β signaling activation in BRCA cells (24). Mechanistically, upon TGF-β stimulation, SMAD4 directly induced 4E-BP1 expression, which interacted with the translation initiation factor eIF4E and decreased CIP2A-BP synthesis. Furthermore, immunoprecipitation assays revealed that the cancerous inhibitor of protein phosphatase 2A (CIP2A) B56γ subunit directly bound to CIP2A-BP (24). Thus, CIP2A-BP can release PP2A activity through competitive binding, which inhibits the PI3K/AKT/NF-κB pathway, followed by the reduction of MMP-2, MMP-9, and SNAI1 expression. Additionally, low CIP2A-BP, but not *LINC00665*, expression in TNBC patients correlated with metastasis and poor overall survival. Hence, TGF-β signaling can lead to TNBC metastasis partially due to the downregulation of CIP2A-BP translation (24).

## Ovarian cancer

Ovarian cancer (OC) is a major cause of lethality related to gynecological tumors with 295,000 new cases and 185,000 deaths annually worldwide (25). Thus, non-coding RNAs emerge not only as novel promising biomarkers for OC prognosis but also as therapeutic targets to prevent OC metastasis and inevitably chemoresistance, which is coupled to tumor recurrence and poor outcome of OC patients (26). Based on available human cancer datasets, higher *cancer susceptibility candidate 15* (*CASC15*) expression correlated with the poor prognosis of OC patients (27). Mechanistically, *CASC15* acted as ceRNA targeting *miR-23b-3p* and *miR-24-3p* and their sequestration cooperatively upregulated the levels of *SMAD3*, triggering the EMT program, which led to cell migration and invasion of OC cells in culture and *in vivo* (27). Another lncRNA expressed at advanced stages of OC patients, which correlated with poor overall survival, progression-free survival, and post-progression survival according to human cancer datasets, was the *prostate cancer-associated transcript 6* (*PCAT6*) (28). Functionally, *PCAT6* increased the proliferation and motility of OC cells in culture and by targeting *miR-143-3p*, as previously reported in pancreatic ductal adenocarcinoma (29). This lncRNA induced the expression of *TGF-*β*-activated kinase 1* (*TAK1/MAP3K7*), which is a serine/threonine kinase of the MAP3K family and critical regulator of TGF-β signaling (28). Additionally, in the context of OC, *PVT1* can possibly regulate the TGF-β pathway by sponging *miR-148a-3p*, which upregulates *AGO1* expression and increases p-SMAD2 and p-ERK1/2 levels (30). Although *PVT1* expression was significantly higher in OC tissues in comparison to non-tumor ovaries, its expression was also correlated with advanced FIGO (International Federation of Gynecology and Obstetrics) stage III-IV, comprising tumor size and lymph-node metastasis, besides poor survival of OC patients. *LINC02323* was also relevant to TGF-β pathway regulation in OC cells by acting as ceRNA for *miR-1343-3p* (31), as also reported in lung adenocarcinoma cells (32), which eventually increased *TGFBR1* levels and, therefore, enhanced TGF-β signaling. Furthermore, *LINC02323* exhibited significantly higher serum levels in OC patients in comparison to normal volunteers, and its function could contribute to OC metastasis. However, how *PCAT6*, *PVT1*, and *LINC02323* expressions were modulated in OC cells was not discussed and remains an open question.

It is established that extracellular vesicles (EVs) carry molecules associated with the TGF-β pathway, including non-coding RNAs (33). In line with this, Yuan et al. claimed that *lncRNA ATB* present in OC-derived EVs could modulate tumorigenesis via sponging the *miR-204-3p*, which downregulates *TGFBR2* expression (34). However, this study had limitations since it did not clarify whether this lncRNA was naturally associated with OC-derived EVs or was found enriched in vesicles due to the isolation method (e.g. force of centrifugation).

## Endometrial cancer

Endometrial cancer (EC) is a fatal malignancy among women globally, and the emerging role of lncRNAs and their dysregulation in EC has been explored in recent studies (35). In this context, overexpression of *MIR210HG* induced Wnt/β-catenin and TGF-β/SMAD3 signaling pathway genes by acting as ceRNA, sponging *miR-337-3p* and *miR-137* to regulate HMGA2 expression, which promoted the expression of EMT-TFs (*SNAI1* and *SNAI2*) and increased EC cell motility (36). Nevertheless, how *MIR210HG* expression was modulated in EC cells was not discussed.

## Head and neck cancer

Head and neck cancer is the seventh most common tumor worldwide, affecting 880,000 new patients and causing 300,000 deaths every year (25). These tumors arise from the mucosa of distinct head and neck topologies, including the oral cavity, pharynx, and larynx, with squamous cell carcinomas (HNSCC) being the most frequent subtype, and due to its higher recurrence, only 30–50% of patients with locally advanced disease survive more than 5 years (37). Dysregulation of lncRNA expression affecting HNSCC tumorigenesis has been reported, providing also new mechanisms toward therapeutic strategies for this highly metastatic tumor (38). Accordingly, *LINC01116* can be highly expressed in HNSCC tissues, and its expression correlated with shorter overall survival and relapse-free survival, based on patient data in the GEO database (39). Additionally, *LINC01116* silencing inhibited invasion of HNSCC cells by blocking EMT, which was in line with a reduction in *SNAI1* and *SNAI2* expressions (39). Another lncRNA that could promote TGF-β signaling, inducing motility of HNSCC cells was the *MIR4435-2HG* (40). *MIR4435-2HG* overexpression led to upregulation of TGF-β1 expression, although exogenous TGF-β1 stimulation had no impact on *MIR4435-2HG* expression. Notwithstanding, mechanistically, how *LINC01116* and *MIR4435-2HG* expressions are regulated, apart from how *LINC01116* could affect the signature of genes related to TGF-β signaling and how *MIR4435-2HG* promoted TGF-β1 upregulation was not investigated in depth.

In tongue SCC (TSCC) tumors, the most prevalent malignancy in the oral cavity, *cancer susceptibility 18* (*CASC18*), acted as a ceRNA to sponge *miR-20a-3p*, enhancing TGF-β2 expression and secretion, which consequently contributed to EMT (41). Higher *CASC18* expression was associated with the status of occult lymph node metastasis in TSCC cohorts, but it was not discussed how its expression could be modulated. Moreover, the lncRNA *SBF2 antisense RNA 1* (*SBF2*-*AS1*) was proposed to sponge *miR*-*302b*-*3p*, upregulating *TGFBR2* expression in laryngeal SCC cells (42). Of note, considering the key role of TGF-β signaling in promoting EMT in laryngeal SCC cells (43), and since *SBF2-AS1* could induce *TGFBR2* expression, it would be expected that this lncRNA could enhance EMT. However, upon knockdown of *SBF2-AS1*, laryngeal SCC cells became more invasive, with higher expression of EMT markers (e.g. vimentin and N-cadherin), which, in turn, is contradictory to the lower levels of *TGFBR2*. Hence, although low *SBF2-AS1* expression was correlated to lymph node metastasis and to advanced clinical stage, the mechanism of *SBF2-AS1* regulating EMT in HNSCC may not involve TGF-β signaling. In a screen for lncRNAs regulated by TGF-β signaling during EMT, the *lnc-PNRC2-1* was the most significantly upregulated lncRNA after TGF-β stimulation for 24 h in nasopharyngeal carcinoma cells (44). Knockdown of *lnc-PNRC2-1* reduced the expression of EMT markers, although the mechanisms of *lnc-PNRC2-1-*modulating EMT and TGF-β signaling remain to be clarified. On the other hand, *EPB41L4A-AS2* is a lncRNA whose expression is attenuated by TGF-β signaling in nasopharyngeal carcinoma cells (45). Overexpression of *EPB41L4A-AS2* reduced the motility of HNSCC cells in culture and *in vivo*, and mechanistically, *EPB41L4A-AS2* bound to YBX1 in the nucleus, to reduce the stability of *SNAI1* mRNA, decreasing EMT progression, while in the cytoplasm, *EPB41L4A-AS2* sponged *miR-107*, promoting *LATS2* expression. Although the mechanism by which *EPB41L4A-AS2* was repressed by TGF-β signaling was not revealed, these findings contributed to novel understanding about lncRNAs modulated by the TGF-β pathway and regulating EMT and metastasis in HNSCC cells.

## Lung cancer

Similar to the previous tumor types, lung cancer remains a major health threat worldwide (11), and cases of lung cancer cell regulation by lncRNAs that link to the biology of TGF-β have been reported. The majority of such reports have analyzed non-small cell lung cancer (NSCLC) cells or patients. These include oncogenic lncRNAs that, via different molecular mechanisms, activate or promote TGF-β signaling. *MIR100HG* can be transcriptionally induced by TGF-β signaling to generate both a lncRNA and processed miRNAs (46). While the function of the encoded miRNAs remains unclear, the full-length *MIR100HG* transcript associated with the RNA binding protein HuR, which simultaneously bound to the *TGFB1* mRNA. This caused its stabilization and, thus, promoted secretion of pro-oncogenic TGF-β1 protein in lung cancer cells (46). The TF FOXP3, which is best known for inducing differentiation of regulatory T cells, can also be misexpressed in NSCLC cells, thus inducing the expression of *LINC01232* that associates directly with the IGF2BP2 protein; IGF2BP2 is an RNA-binding protein that associated with and stabilized the *TGFBR1* mRNA, thus causing an indirect enhancement of pro-oncogenic TGF-β signaling in the lung cancer cells (47). *LINC00511*, which is selectively overexpressed in NSCLC patients, can function as a ceRNA for *miR-98-5p*, another negative regulator of *TGFBR1* mRNA expression, thus making *LINC00511* an activator of pro-oncogenic TGF-β signaling in NSCLC (48). The *small nucleolar RNA host gene 3* (*SNHG3*) can be highly expressed in NSCLC possibly due to the transcriptional activation of its gene by E2F1 and belongs to the general class of ceRNAs that sponge miRNAs (49). Since chemical inhibitors against TGFβRI blocked the proliferative and migratory actions of overexpressed SNHG3, this lncRNA was postulated to activate TGF-β signaling (49). However, chemical inhibition of JAK2 signaling also blocked the proliferative and migratory actions of overexpressed SNHG3, implicating also interleukin-6 signaling. In this case, the ceRNA function of *SNHG3* has not been established, and identifying miRNAs that inhibit TGF-β or interleukin-6 signaling maybe a relevant mechanism to examine in the future. A very similar mechanism involves *MIR4435-2HG* that is highly expressed in NSCLC, which was bioinformatically predicted to sponge distinct miRNAs that can target the *TGFB1* mRNA, thus indirectly enhancing TGF-β signaling that was required for lung cancer cell proliferation and migration (50).

LncRNAs can also regulate TGF-β signaling at the level of SMAD protein function. The *non-coding RNA activated by DNA damage* (*NORAD*) associated with importin-β1, which also bound to SMAD3 and mediated its nuclear translocation in lung adenocarcinoma cells (51). Thus, a subset of genes regulated by TGF-β received an enhanced signal, and these included genes of the EMT program that facilitate lung cancer cell migration (51). Induction of expression of *AC026904.1* by TGF-β signaling correlated with high expression of this lncRNA in metastatic lung cancer and *AC026904.1* further induced transcription of SNAI2 via a yet uncharacterized molecular mechanism (52). *AC026904.1* possibly provides a molecular link between lung cancer metastasis and TGF-β/SNAI2 expression during EMT. Also implicated in the EMT process is the TGF-β-inducible *LINC00273*, whose ceRNA function sponged a well-established negative regulator of EMT, the *miR-200a-3p*, and directly limited the expression of ZEB1, a mechanism that explains a strong pro-metastatic action of *LINC00273* (53). At the end stages of metastasis, circulating lung cancer cells can colonize the brain, and before this, they must pass through the blood–brain barrier of capillaries. Such circulating lung cancer cells responded to TGF-β signaling by inducing secretion of EVs that carry the lncRNA *lnc-MMP2-2*, which acted as a ceRNA for *miR-1207-5p* (54). One of the molecular targets of *miR-1207-5p* can be the mRNA for the EPB41L5 adaptor protein that participates not only in the Crumbs complex that organizes epithelial tight junctions but also in invadopodia associated with integrins. The proposed model suggests that metastatic cells, by secreting EVs in response to TGF-β, transmit *lnc-MMP2-2* to endothelial cells that eventually stabilize EPB41L5, causing a more permeable blood–brain barrier and facilitating the metastatic colonization (54).

While all previous lung cancer examples contribute to activation or enhancement of TGF-β signaling, lncRNAs can also limit or inhibit this signaling pathway. Accordingly, *SMAD3-associated long non-coding RNA* (*SMASR*) can be transcriptionally repressed by SMAD signaling (55). SMASR interacted directly with SMAD3, and their complex associated with the chromatin of the *TGFBR1* gene causing its repression, thus limiting TGF-β signaling and lung cancer EMT (55). An independent lncRNA-mediated mechanism operated at the TGFβRI protein stability level. *LITATS1* can be under-expressed in NSCLC tissue relative to normal lung epithelia and molecularly associated with TGFβRI and its E3 ubiquitin ligase SMURF2 (56). This RNA-mediated protein complex caused degradation of TGFβRI, thus limited TGF-β signaling, whereas tumor cells in which *LITATS1* was downregulated presented enhanced TGF-β signaling and EMT (56). Additional lncRNAs acting at the chromatin level include the *TGFB2 antisense RNA 1* (*TGFB2-AS1*), whose expression can be induced by TGF-β in lung adenocarcinoma and other cell types. This lncRNA could interact with the polycomb repressor complex 2 adaptor protein embryonic ectoderm development (EED), thus blocking full responsiveness of genes to TGF-β signaling (57). A similar mechanism, but via interaction between *TGFB2-AS1* and the SWI/SNF chromatin remodeling protein SMARCA4, has been shown to mediate partial blocking of gene expression to TGF-β signaling, but in the context of BRCA cells (58). Whether lncRNA partners with distinct but functionally equivalent proteins regulate TGF-β signaling is an interesting concept that needs to be explored deeper.

## Pancreatic adenocarcinoma

The 5-year survival rate for pancreatic adenocarcinoma (PDAC) remains below 8%, corresponding to the seventh leading cause of cancer-related death worldwide. LncRNAs, such as *HOTAIR*, *HOTTIP*, *MALAT1*, *H19*, *PVT1*, *GAS5*, *MEG3*, and *ENST00000480739*, have been linked to modulation of growth and invasion of PDAC cells (59). Additionally, *MIR31HG* (or *long non-coding HIF-1α co-activating RNA – lncHIF-CAR*) can be upregulated by TGF-β signaling in PDAC cells (60). The levels of *MIR31HG* were further correlated with the EMT gene signature in PDAC patient datasets, and its higher expression was associated with worse disease-free survival in patients. Moreover, *MIR31HG* silencing downregulated TGF-β-induced EMT, but the questions about how TGF-β modulated *MIR31HG* expression and how mechanistically *MIR31HG* promoted the TGF-β-induced EMT were not addressed (60). Another lncRNA that increases cell proliferation and invasion of PDAC cells and might play a role in TGF-β signaling was the X-inactive specific transcript (*XIST*) (61). This lncRNA sponged *miR-141-3p*, a negative regulator of *TGFB2* transcripts. The *highly upregulated in liver cancer* (*HULC*) is another lncRNA, whose expression can be induced by TGF-β in PDAC cells and in PDAC-derived EVs (62). Since *HULC* silencing via siRNA or *microRNA-133b* decreased PDAC cell invasion by inhibiting the EMT, the authors proposed that *HULC* could be transferred to neighboring cells by PDAC-EVs, in order to promote EMT, although the mechanism of how *HULC* can modulate EMT in PDAC cells remains not fully understood. Furthermore, the enrichment of *HULC*-associated EVs present in the serum of PDAC patients showed good predictive power for discriminating PDAC patients, suggesting a novel tool for the early diagnosis of human PDAC (62). Nonetheless, the *long intergenic non-coding RNA 261* (*LINC00261*) was downregulated upon TGF-β stimulation in PDAC cells (63). The TF forkhead box protein A2 (FOXA2) directly bound to the *LINC00261* promoter, inducing the expression of this nuclear lncRNA. It has been shown by others that TGF-β signaling transcriptionally represses FOXA2 expression (64), which suggested that TGF-β could decrease *LINC00261* expression by downregulating *FOXA2* levels, although whether this could happen through a SMAD-dependent or -independent mechanism remains to be characterized. Moreover, a regulatory network between FOXA2 and *LINC00261* regulated E-cadherin expression, which is an important epithelial cell adhesion protein (63). In view of this, the authors proposed that the repression of FOXA2 and *LINC00261* expression by TGF-β facilitated EMT by triggering the loss of E-cadherin-dependent adherence junctions and, consequently, enhanced invasiveness of PDAC cells.

## Hepatocellular carcinoma

Hepatocellular carcinoma (HCC) is the major subtype of liver cancer and one of the most frequent and lethal malignancies worldwide. To better understand HCC tumorigenesis, several studies reported lncRNAs modulating malignant phenotypes in HCC cells (e.g. evading apoptosis or enhancing cell proliferation and invasion) through their functional activity associated with DNA, RNA, or proteins (65). The mechanisms of lncRNAs and TGF-β in HCC initiation and development have been previously reviewed (66). We therefore discuss mainly more recent reports. The lncRNA *nicotinamide nucleotide transhydrogenase-antisense RNA1* (*NNT-AS1*) could impair CD4^+^ T cell infiltration via the activation of TGF-β signaling in HCC cells (67). The authors proposed that *NNT-AS1* contributed positively to *TGFB1*, *TGFBR1*, and *SMAD5* expressions in HCC cells, although the mechanism of such regulation was not described. Additionally, *NNT-AS1* levels were significantly increased in HCC tissues in comparison to normal tissues, and the elevated *NNT-AS1* levels were correlated with poor patient overall survival. Similarly, the expression of *maternally expressed gene 8* (*MEG8*) was increased in HCC and correlated with the poor prognosis of HCC patients (68). Interestingly, the authors suggested that *MEG8* enhanced 14-3-3ζ expression by acting as a ceRNA of *miR-367-3p*. Thus, based on other studies (69), 14-3-3ζ might suppress TGFβR1 degradation, thereby enhancing TGF-β signaling, which provided HCC cells overexpressing *MEG8* a higher ability to proliferate, migrate, and invade. In the context of ceRNA function in HCC, *SET-binding factor 2 antisense RNA1* (*SBF2-AS1*) bound to the *miR-361-5p*, which, in turn, negatively regulated *TGFB1* expression, affecting HCC proliferation and migration (70). Whether the expression of *NNT-AS1* and *SBF2-AS1* could be modulated by TGF-β signaling remains unknown.

As noted in PDAC (63), *Linc00261* was also downregulated by TGF-β1 in HCC cells, and its downregulation facilitated EMT and stemness by increasing SNAI2 and ZEB1, OCT4, and SOX2 expressions (71). Mechanistically, the authors claimed that *Linc00261* induced SMAD3 ubiquitin-proteasome-mediated degradation, which consequently led to decreased amounts of phosphorylated SMAD3. However, the authors did not demonstrate whether *Linc00261* modulated SMAD3 directly or indirectly, e.g. via binding to SMAD3 or not. In addition, possible effects of *Linc00261* on SMAD2 or non-SMAD signaling were not investigated. Nevertheless, the in-culture and *in vivo* evidence that *Linc00261* overexpression could inhibit TGF-β signaling in HCC cells is strong (71).

## Colorectal cancer

CRC is the second leading cause of cancer death in men and women combined worldwide (11), and lncRNAs are considered important players for the molecular mechanisms driving the initiation, progression, and metastasis of CRC (72). Similar to BRCA (14) and HCC (13) patients, high *lncRNA‑ATB* expression was associated with poor overall survival of CRC patients, besides advanced TNM stage, comprising tumor extent (T), spread to lymph node (N), or metastasis (M) to distant sites (73, 74). Notably, different studies observed that *lncRNA‑ATB* promoted CRC cell growth and motility through its role as a ceRNA to *miR‑141‑3p* (74) or to *miR-200c*, which, in turn, targeted *CDK2* expression (73). These findings are in line with the demonstration that *lncRNA-ATB* knockdown also inhibited CRC growth, besides colony and sphere formation (75). Furthermore, *lncRNA-ATB* could inhibit β‑catenin expression, an oncogenic factor in CRC (75). Another lncRNA highly expressed in CRC patients and induced by TGF-β is the *taurine upregulated gene 1* (*TUG1*), whose knockdown decreased EMT, followed by the reduction of CRC cell motility in culture and inhibition of CRC lung metastasis *in vivo* (76). Mechanistically, *TUG1* appeared to act downstream of TGF-β signaling by modulating the expression of *TWIST1*, as the silencing of *TUG1* decreased the levels of *TWIST1* induced by TGF-β in CRC cells. Nevertheless, how TGF-β signaling induced *TUG1* expression, via SMADs or not, remains open for debate. An additional ceRNA affecting firmly TGF-β signaling is *LOC646329-variant D*, whose overexpression suppressed CRC progression through sponging *miR-29b-1*, which is harbored at the third intron of *LOC646329*, followed by the upregulation of *SMAD3* and *p21* levels, while *cyclinD1* was downregulated, shifting a high percent of the CRC cell population to the G1 phase (77). Furthermore, the expression of the lncRNA *CTBP1 divergent transcript* (*CTBP1-DT* or *CTBP1-AS2*) was associated with worse overall survival of CRC patients and positively correlated with *TGFB1*, *SMAD2*, and *SMAD3* in both colon and rectal adenocarcinoma patients (78). *CTBP1-AS2* enhanced TGF-β signaling, in addition to affecting CRC cell proliferation and invasion, by sponging and inhibiting *miR-93-5p* function, which potentially targets *TGFB1* mRNA. However, whether the other *LOC646329* variants could impact *miR-29b-1* levels and how the expression of *CTBP1-AS2* and *LOC646329* is modulated by TGF-β remains poorly explored.

*LINC00941*, whose expression is induced by TGF-β, promoted EMT in CRC cells by directly binding the MH2 domain of SMAD4, as shown by RIP and RNA pulldown assays (79). *LINC00941* competed with the β-transducin repeats-containing protein (β-TrCP) E3 ubiquitin ligase to bind SMAD4, and this competition prevented SMAD4 degradation, thus activating TGF-β signaling, which induced the expression of cell invasion and metastasis genes (e.g. vimentin, fibronectin, and TWIST1), while decreased the levels of the main invasion suppressors E-cadherin and ZO-1 in CRC cells (79). Moreover, higher expression of *LINC00941* was associated with poor prognosis of CRC patients (79). Yet, in the context of CRC, the expression of lncRNA *small nucleolar RNA host gene 10* (*SNHG10*) was upregulated in tumor tissues and associated with poor prognosis of CRC patients (80). Furthermore, since the activation of TGF-β signaling might affect the sorting of molecular cargo from tumor-derived EVs (33), CRC cells (SW480) were kept in the presence of TGF-β for 72 h, and upon EV isolation followed by RNA sequencing, high enrichment of *SNHG10* was found relative to EVs derived from non-stimulated cells (80). Functionally, EV-associated *SNHG10* promoted CRC growth and suppressed NK cells *in vivo* by upregulating inhibin-βC (INHBC) expression in NK recipient cells, but how mechanistically TGF-β signaling affected the sorting of *SNHG10* to CRC-derived EVs was not addressed.

## Bladder cancer

The repertoire of lncRNAs correlated with the occurrence and development of bladder cancer (BC), a urological malignancy associated with high mortality and morbidity, includes *UCA1*, *HOTAIR*, *MEG3*, *H19*, *GAS5*, and *MALAT1* (81). *PLAC2* acted as a tumor suppressor lncRNA in BC since its expression was downregulated in tumors when compared to non-cancer tissues. By unknown mechanism(s), *PLAC2* induced *miR-663* expression, which targets *TGFB1*, decreasing invasion of BC cells (82). Moreover, *MIR497HG* and its two harbored miRNAs, *miR-497* and *miR-195*, were also downregulated in BC cells (83). Interestingly, the function of *MIR497HG* was related to its harbored miRNAs, which coordinately suppressed multiple key components in the Hippo/YAP and TGF-β pathways, particularly attenuating the interaction among YAP and SMAD3, thus affected cell growth and invasion in culture. In addition, the E2F4 TF was critical to repress *MIR497HG* transcription in BC cells. On the other hand, *Linc02470* was identified in the lnCAR database as one of the most upregulated lncRNAs expressed during BC initiation and progression (84). In the context of TGF-β signaling, *Linc02470* directly targeted the *miR-143-3p*, promoting *SMAD3* expression, which consequently induced SNAI1, SNAI2, ZEB2, vimentin, and N-cadherin expression. Hence, this lncRNA plays a role in promoting the EMT and increasing motility of BC cells. Furthermore, *cancer susceptibility candidate 9* (*CASC9*) was also significantly upregulated in BC cells when compared to normal bladder tissues (85). Functionally, *CASC9* acted as a ceRNA for *miR‑758‑3p*, a miRNA which repressed *TGFB2* expression. Thus, by enhancing TGF‑β2 levels in BC cells, *CASC9* promoted EMT and invasion (85). Notwithstanding, the mechanisms representing how *Linc02470* and *CASC9* expressions were induced or the repression of *PLAC2* in BC cells occurred were not explored, necessitating further studies. *LINC01451* can be highly expressed in BC in comparison to normal tissues, and its high expression correlated with poor prognosis of patients (86). Upon the pull-down assay of biotinylated *LINC01451*, direct interaction of *LINC01451* with LIN28A and LIN28B (both being RNA binding proteins) was found, which promoted BC cell proliferation, invasion, and metastasis. Since *TGFBRI* and *TGFBRII* expressions were decreased in the absence of *LINC0145* in BC cells, a reduction of EMT induced by TGF-β signaling was noted, which subsequently abrogated BC progression (86). Nevertheless, how *LINC01451* relying on LIN28A and LIN28B modulated the TGF-β receptors to promote EMT remains unclear.

## Prostate cancer

Prostate cancer, a serious tumor type in men, makes no exception to the importance of lncRNA-mediated functions that relate to different hallmarks of cancer (11). Although TGF-β signaling does not seem to regulate expression of *NCK1-AS1*, this lncRNA was highly expressed in prostate carcinomas and underexpressed in normal or even benign hyperplastic prostate tissue and seems to activate *TGFB1* mRNA and TGF-β signaling via as yet unknown molecular mechanism (87). In contrast, the *MIR100HG* gene that was described in the lung cancer section was transcriptionally induced by TGF-β and formed a feed-forward signaling loop by facilitating *TGFB1* mRNA stabilization and growth factor secretion by prostate cancer cells (46). Similarly, *prostate cancer-associated transcript-7* (*PCAT7*) expression was induced by TGF-β signaling via a SMAD3 and SP1 TF-dependent mechanism (88). This mechanism seems to mark primary prostate carcinomas with a strong potential for metastasis to the bone. Furthermore, one possible function of *PCAT7* is to serve as ceRNA for *miR-324-5p*, thus prohibiting access of this miRNA to one of its predicted target mRNAs, *TGFBR1*. Accordingly, high expression of *PCAT7* in prostate carcinoma will sponge *miR-324-5p* and enhance *TGFBR1* expression and TGF-β signaling, which can feed back to the gene and enhance its expression in cells that prepare for metastasis (88). An exactly similar scenario has been described for the *small nucleolar RNA host gene 3* (*SNHG3*), which enhanced *TGFBR1* mRNA expression by sponging away the negative regulation exerted by *miR-214-3p* (89). An equivalent ceRNA-mediated mechanism seems to involve regulation of *TGFBR2* and involves the *small nucleolar RNA host gene 16* (*SNHG16*) lncRNA, which is highly expressed in prostate carcinoma tissue and cells (90). High *SNHG16* expression seems to sponge and inactivate *miR-373-3p*, which can downregulate the *TGFBR2* mRNA. Thus, in prostate cancer cells with high *SNHG16* expression, TGFβRII protein levels increased, causing enhanced TGF-β signaling that facilitated cancer invasiveness (90). Keeping in the same mode of action, *DANCR* expression correlated with Gleason score and prostate-specific antigen levels in the serum of prostate cancer patients (91). *DANCR* can sponge different miRNAs, among which, *miR-214-5p* has been linked to the negative regulation of TGF-β signaling. Both TGF-β1 ligand and TGFβRI receptor protein levels along with SMAD3 signaling can be reduced by *miR-214-5p* and enhanced by the antagonistic *DANCR* (91). This mechanism may require deeper understanding along with the fact that *miR-214-3p* (89), the miRNA generated by the same pre-miRNA as *miR-214-5p*, has been proposed to have the opposite effect on prostate cancer cell *TGFBR1* levels, as described earlier. Thus, lncRNAs in prostate cancer cells can mediate regulation of the ligand and of both TGF-β receptors.

LncRNAs that can negatively regulate TGF-β signaling have also been reported in prostate cancer. Negative regulation at the SMAD protein level can be relevant in prostate cancer cells, with *LINC00707* being an example of double negative feedback regulation (92). TGF-β signaling via SMAD3 and mitogen activated protein kinases (MAPKs) displaced the TF KLF6 from the *LINC00707* gene, thus downregulating its expression. *LINC00707* also bound to SMAD proteins and sequestered them to the cytoplasm where *LINC00707* resides, thus limiting the output of TGF-β signaling in prostate cancer cell cultures (92).

## Glioblastoma

The brain malignancy glioblastoma (GBM) is considered as one of the most aggressive and lethal human tumors. Of importance, several lncRNAs were described modulating molecular and cellular processes, which affected GBM heterogeneity and treatment resistance (93). For instance, TGF-β/SMAD signaling induced the expression of *lncRNA-MUF* (*LINC00941*) in GBM cells (94). Moreover, *lncRNA-MUF* could upregulate the effect of TGF-β inducing *CAPRIN2* expression in a *cis* manner, apart from sponging *miR-34a*, which targets *SNAI1*. Nevertheless, although in the absence of *lncRNA-MUF*, the levels of SMAD2/3 phosphorylation, the mRNA levels of *vimentin*, *connective tissue growth factor*, and *MYC*, and the invasiveness phenotype were reduced in GBM cells. The mechanism by which this lncRNA impacted SMAD2/3 activation to promote TGF-β signaling was not clarified (94). It is also important to mention that *LncRNA-MUF* expression induced by TGF-β in GBM cells was correlated with temozolomide (TMZ) resistance (94). Additionally, two other lncRNAs (*H19* and *HOXD-AS2*) that were induced by TGF-β1 through SMAD signaling were associated with TMZ resistance in GBM. The interaction of *H19* and *HOXD-AS2* with the K-homology splicing regulatory protein (KSPR) prevented the binding of this protein to the primary *miR-198*, due to a weaker association between KSRP and Drosha/Dicer complexes, leading to reduced *miR-198* expression. Consequently, O6-methylguanine methyltransferase, which is a target of *miR-198*, was elevated, driving TMZ resistance of GBM cells because of its reverse effect on DNA alkylation by removing the methyl groups from TMZ-induced O6-methyguanine lesions (95, 96). Another lncRNA whose expression was correlated with the worst prognosis of GBM patients was the *MIR4435-2HG* (97). The relevance of this lncRNA to TGF-β signaling is represented by its role in culture as a ceRNA binding to *miR-1224-5p*, which has *TGFBR2* transcripts as a target. Finally, *miR-133b-3p*, which possibly targets *TGFB1* transcripts, bound directly to the *Lnc HOXA transcript antisense RNA, myeloid-specific 1* (*HOTAIRM1*) in high-grade gliomas and in malignant transformed fibroblasts (98). Thus, by competing with *miR-133b-3p*, *HOTAIRM1* increased *TGFB1* expression and TGF-β signaling in gliomas. Nevertheless, how the expressions of *MIR4435-2HG* and *HOTAIRM1* were regulated and how *miR-133b-3p* regulated *TGFB1* mRNA in GBM cells remain open questions.

## Conclusions

The large number of ncRNA genes that are widespread in the human genome suggest that the biological impact of such genes in diverse aspects of human biology will continue being uncovered actively in the near future and will also continue permeating cancer biology and TGF-β signaling. The examples presented in this article (also summarized in Table 1) are not exhaustive, yet they represent mechanisms of positive and negative signaling of the TGF-β pathway. We did not review every tumor type (e.g. hematopoetic tumors) but selected those types where the evidence for lncRNA functions linked to TGF-β signaling is stronger. Other TGF-β family pathways (activin, BMP, growth and differentiation factor (GDF), and Müllerian inhibiting substance (MIS)) are relevant to consider from a lncRNA perspective, and these are not discussed here. As explained earlier, the mechanisms may entail regulation at the ligand, receptor, SMAD, and signaling via alternative pathways, and also regulation at the chromatin-transcriptional level. The ceRNA mechanism appears dominant, yet the multifunctionality of every single miRNA generates anxiety about the specificity of the proposed mechanism and the reliability of the mechanism. This complexity always brings in mind that a single lncRNA or miRNA cannot be highly specific only for TGF-β signaling. Thus, modern studies do have to consider analyses of some of the alternative targets of these RNA molecules. Throughout our article, we attempted to provide some critical views on specific mechanistic models and even more around the lack of such mechanisms, in studies that correlate lncRNA expression to that of TGF-β pathway genes, EMT genes, and to the survival of patients. The latter is an abundant feature in lncRNA studies, facilitated by the availability of databases, and always suffers from the lack of validation of these predictions using cancer patient specimen and complementary (to transcriptomic) techniques by the investigators. Despite such criticism, the lncRNA field evolves rapidly, and the RNA-based components that complement key signaling events in TGF-β signal transduction enrich our knowledge and provide new ideas about molecular links that can generate explanations to previously unresolved problems of signal transduction in the context of human cancer.

**Table 1:** Regulatory network of TGF-β signaling and lncRNAs in tumors.

LncRNA	Mechanism of action	Cancer type	Ref.
**Oncogenic action**			
*LncRNA-ATB*	ceRNA of several miRNAs: *miR-204-3p*; *miR‑141‑3p*; *miR-200c* (targeting CDK2)	Breast; Ovarian; Colorectal	(13–15, 34, 73, 74)
*Smyca*	Increases SMAD3/4 complex stability	Breast	(16)
*TMPO-AS1*	Enhances *TGFBR1* and *TGFBR2* mRNA expression	Breast	(20)
*LETS1*	Facilitates SMAD7 polyubiquitination and its degradation through NFAT5 complex and NR4A1 regulation	Breast	(22)
*CASC15*	ceRNA of *miR-23b-3p* and *miR-24-3p* to enhance *SMAD3* expression	Ovarian	(27)
*PCAT6*	ceRNA of *miR-143-3p* to enhance *TAK1*/ *MAP3K7* mRNA expression	Ovarian	(28, 29)
*PVT1*	ceRNA of *miR-148a-3p* to enhance *AGO1* mRNA expression	Ovarian	(30)
*LINC02323*	ceRNA of *miR-1343-3p* to enhance *TGFBR1* mRNA levels	Ovarian; Lung	(31, 32)
*MIR210HG*	ceRNA of *miR-337-3p* and *miR-137* to enhance *HMGA2* expression	Endometrial	(36)
*LINC01116*	Enhances *SNAI1* and *SNAI2* expression	Head and neck	(39)
*MIR4435-2HG*	Upregulates TGF-β1 expression	Head and neck	(40)
*CASC18*	ceRNA of *miR-20a-3p*, increasing *TGFB2* mRNA expression	Head and neck	(41)
*Lnc-PNRC2-1*	Silencing decreases the expression of EMT markers	Head and neck	(44)
*LINC00941* or *lnc RNA-MUF*	Induces CAPRIN2 expression in a cis manner and sponges miR-34a to promote *SNAI1* expression	Glioblastoma	(94)
*MIR4435-2HG*	ceRNA of *miR-1224-5p*, enhancing *TGFBR2* mRNA levels	Glioblastoma	(97)
*HOTAIRM1*	ceRNA of *miR-133b-3p*, increasing *TGFB1* mRNA expression	Glioblastoma	(98)
*Linc02470*	ceRNA of *miR-143-3p*, increasing *SMAD3* mRNA expression	Bladder	(84)
*CASC9*	ceRNA of *miR-758-3p*, increasing *TGFB2* mRNA expression	Bladder	(85)
*XIST*	ceRNA of *miR-141-3p*, increasing *TGFB2* mRNA expression	Pancreatic adenocarcinoma	(61)
*NNT-AS1*	Contributes positively to *TGFB1*, *TGFBR1*, and *SMAD5* mRNA expression	Hepatocellular carcinoma	(67)
*MEG8*	ceRNA of *miR-367-3p*, increasing *14-3-3ζ* expression, which suppresses TGFβR1 degradation	Hepatocellular carcinoma	(68)
*TUG1*	Induces TWIST1 expression	Colorectal	(76)
*CTBP1-AS2*	ceRNA of *miR-93-5p*, increasing *TGFB1* mRNA expression	Colorectal	(78)
*LINC00941*	Binds to SMAD4 enhancing the protein stability by competing with β-TrCP	Colorectal	(79)
*MIR100HG*	Binds to HuR, which promotes *TGFB1* mRNA stabilization, followed by secretion of pro-oncogenic TGF-β1	Lung; Prostate	(46)
*LINC01232*	Associates with IGFBP2 to promote *TGFBR1* mRNA stability	Lung	(47)
*LINC00511*	ceRNA of *miR-98-5p*, increasing *TGFBR1* mRNA levels	Lung	(48)
*NORAD*	Associates with importin-β1 facilitating SMAD3 nuclear translocation	Lung	(51)
*LINC00273*	ceRNA of *miR-200a-3p*, increasing *ZEB1* levels	Lung	(53)
*lnc-MMP2-2*	ceRNA of *miR-1207-5p*, increasing *EPB41L5* levels	Lung	(54)
*PCAT7*	ceRNA of *miR-324-5p*, increasing *TGFBR1* levels	Prostate	(88)
*SNHG3*	ceRNA of *miR-214-3p*, increasing *TGFBR1* levels	Prostate	(89)
*SNHG16*	ceRNA of *miR-373-3p*, increasing *TGFBR2* levels	Prostate	(90)
DANCR	ceRNA of *miR-214-5p*, increasing *TGFB1*, *TGFBR2*, and *SMAD3* levels	Prostate	(91)
**Tumor suppressor**			
*TUSC7*	ceRNA of *miR-1224-3p*, repressing *TGFBR2* levels	Breast	(18)
*ADAMTS9-AS2*	Interacts with RPL22 to decrease SMAD2 expression	Breast	(17)
*LINC00665*	Translates to the CIP2A peptide, which inhibits the PI3K/AKT/NF-κB pathway	Breast	(24)
*SBF2-AS1*	ceRNA of *miR-302b-3p* and *miR-361-5p*, which decrease *TGFB1* mRNA levels	Head and neck; Pancreatic adenocarcinoma	(42, 70, 99)
*EPB41L4A-AS2*	Interacts with YBX1 reducing the stability of *SNAI1* mRNA and sponges *miR-107* to promote *LATS2* expression	Head and neck	(45)
*PLAC2*	Induces *miR-663* expression, which targets *TGFB1* mRNA	Bladder	(82)
*MIR497HG*	Attenuates YAP/SMAD3 complex	Bladder	(83)
*LINC00261*	Network between FOXA2 and *LINC00261* regulates E-cadherin expression and induces SMAD3 ubiquitin-proteasome-mediated degradation	Pancreatic adenocarcinoma; Hepatocellular carcinoma	(63, 71)
*LOC646329-variant D*	ceRNA of *miR-29b-1*, increasing *SMAD3* and *p21* expression	Colorectal	(77)
*SMASR*	Interacts directly with SMAD3 repressing SMAD signaling	Lung	(55)
*LITATS1*	Associates with TGFβRI and its E3 ubiquitin ligase SMURF2, inducing the degradation of TGFβRI	Lung	(56)
*TGFB2-AS1*	Interacts with the polycomb repressor complex 2 adaptor protein EED, blocking TGF-β signaling responses	Lung	(57)
LINC00707	Sequesters SMAD proteins to the cytoplasm	Prostate	(92)

TGF-β, transforming growth factor β; IncRNA, long non-coding RNAs.
